# Characteristics of a plastic scintillation detector in photon beam dosimetry

**DOI:** 10.1002/acm2.14209

**Published:** 2023-11-20

**Authors:** Indra J. Das, Jooyoung J. Sohn, Sara N. Lim, Bishwambhar Sengupta, Marcos Feijoo, Poonam Yadav

**Affiliations:** ^1^ Department of Radiation Oncology Northwest Memorial Hospital Northwestern University Feinberg School of Medicine Chicago Illinois USA; ^2^ Blue Physics LLC Lutz Florida USA

**Keywords:** characterization, photon beam, plastic scintillating detector, small field dosimetry

## Abstract

**Background:**

Plastic scintillating detectors (PSD) have gained popularity due to small size and are ideally suited in small‐field dosimetry due to no correction needed and hence detector reading can be compared to dose. Likewise, these detectors are active and water equivalent. A new PSD from Blue Physics is characterized in photon beam.

**Purpose:**

Innovation in small‐field dosimetry detector has led us to examine Blue Physics PSD (BP‐PSD) for use in photon beams from linear accelerator.

**Methods:**

BP‐PSD was acquired and its characteristics were evaluated in photon beams from a Varian TrueBeam. Data were collected in a 3D water tank. Standard parameters; dose, dose rate, energy, angular dependence and temperature dependence were studied. Depth dose, profiles and output in a reference condition as well as small fields were measured.

**Results:**

BP‐PSD is versatile and provides data very similar to an ion chamber when Cerenkov radiation is properly accounted. This device measures data pulse by pulse which very few detectors can perform. The differences between ion chamber data and PSD are < 2% in most cases. The angular dependence is a bit pronounces to 1.5% which is due to PSD housing. Depth dose and profiles are comparable within < 1% to an ion chamber. For small fields this detector provides suitable field output factor compared to other detectors and Monte Carlo (MC) simulated data without any added correction factor.

**Conclusions:**

The characteristics of Blue Physics PSD is uniquely suitable in photon beam and more so in small fields. The data are reproducible compared to ion chamber for most parameters and ideally suitable for small‐field dosimetry without any correction factor.

## 1 INTRODUCTION

Active detectors are preferred due to real‐time dose measurements. However, they should meet certain characteristics such as tissue equivalency, stable and signal reproducibility, dose linearity, dose rate and energy independency. Additionally, they should have minimum angular and temperature dependence. Plastic scintillation detector (PSD) meets most of these characteristics and can represent a valuable tool for small‐field dosimetry due to its small size, portability, energy independence, and tissue equivalence. When properly calibrated and used in conjunction with appropriate signal processing techniques, PSDs offer high‐resolution dose measurements for a wide range of clinical applications, as shown by Beddar et al.^1^, ^2^ The success of PSD led to the marketing of several PSD with unique niche that have been elaborated as a detector of choice as it does not have any perturbation and does not require correction factors in small fields.^3^, ^4^, ^5^, ^6^, ^7^, ^8^, ^9^, ^10^ There are several other PSDs that have inorganic or organic dopant devices showing similar high‐resolution results without correction.^11^, ^12^

Most PSDs consist of a transparent plastic base, typically made of polystyrene, that is, doped with a scintillating material, such as fluor or anthracene. The scintillating material interacts with ionizing radiation, producing photons that are collected and converted into a measurable electrical signal. This process enables the PSD to rapidly measure high‐resolution dose distributions in small fields. In doing so, the fiber also collects Cerenkov radiation, which is subtracted based on manufacturer's design. One key advantage of PSDs is their small size and portability, allowing for easy integration into a variety of dosimetric systems. Modern PSD systems are also quite robust and durable, making them ideal for use in clinical environments. Additionally, they have negligible energy dependence and are nearly tissue‐equivalent, allowing them to provide accurate measurements in a variety of clinical conditions.

When using PSDs for small‐field dosimetry, one must take into consideration the factors that may affect their performance. For example, the size and shape of the PSD can impact dose measurements in small fields due to the increased likelihood of photon scatter. Additionally, the energy response of the PSD must be carefully calibrated to ensure that it measures dose accurately across the energy spectrum of the radiation source. Finally, the use of appropriate signal processing techniques is essential to ensure accurate dose measurements and minimize interference from noise sources mainly Cerenkov radiation.^13^ When properly calibrated and used in conjunction with appropriate signal processing techniques, PSDs offer high‐resolution dose measurements for a wide range of clinical applications.

Among many vendors, the Blue Physics Plastic Scintillation Detector (BP‐PSD) is a new system, whose characteristics were recently published in MR‐Linac.^12^ MR‐Linac is a new technology that combines MRI unit and a linear accelerator on a single gantry.^14^ These machines differ in many ways to the standard linear accelerator, but primarily they operate in magnetic field, with nonstandard source‐to‐axis distance (90‐140 cm) unlike 100 cm for standard machines. Additionally, MR‐Linacs are flattening filter‐free (FFF) design and a single energy and dose rate. Due to Lorenz force associated with magnetic field, dosimetry is very complex, specially the electron return effect.^15^, ^16^ Ferrer et al.^12^ provided dosimetric data using BP‐PSD on Elekta Unity MR‐Linac that has 7 MV photon beam and 1.5 T magnetic field. However, there is no published data for standard linear accelerators which is backbone of the radiation treatment. Additionally, small fields are essential in most advanced treatment techniques where a PSD has been advocated.^9^, ^10^ BP‐PSD has not been evaluated in regular photon beams which is investigated for photon beams from a Varian TrueBeam. A complete detector characterization was evaluated for pulse‐by‐pulse dosimetry, dose and dose rate linearity, energy dependence, angular dependence (due to housing), thermal response and more importantly, the small field output factor, and compared with other detectors on a TrueBeam linear accelerator.

## 2 MATERIALS AND METHODS

**FIGURE 1:**
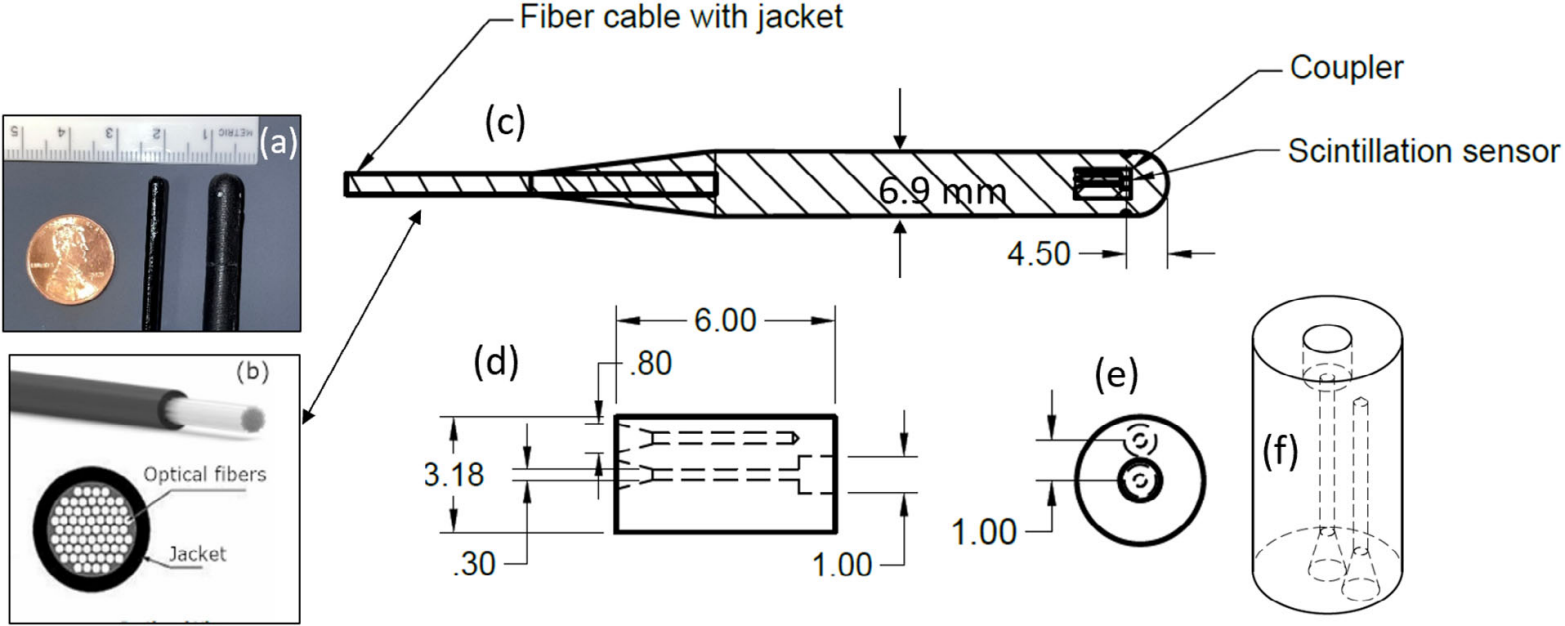
Schematic of the BP‐PSD. (a) two set of detectors, thin and thick. (b) Fiber optics casing that is part of the detector as shown in (c) of the thick detector that resembles thimble ion chamber, (d) cross‐section of the two fibers, one doped scintillator and other for Cerenkov, (e) shows the axial view of the detector dark color in center and Cerenkov fibers anterior to the detector and (f) longitudinal view of detector and Cerenkov fiber.

The BP‐PSD model 10 system consists of several parts: a PSD, transport optical fibers, a removable cartridge, an acquisition unit box, and a single‐board computer with Blue Physics software (BlueSoft) to visualize and analyze the data in real‐time. Figure 1 describes the various components of the BP‐PSD detector system that consists of signal and Cerenkov subtraction fiber. The PSD has a cylindrical shape of 1 mm diameter and 1 mm length, giving it a sensitivity volume of 0.785 mm^3^. The PSD is coupled to one of the transport plastic optical fibers, which has a diameter of 0.25 mm and is 20 m long. This fiber transports the light generated by the PSD to the removable cartridge, located outside the bunker.

The removable cartridge contains all the optical coupling of the transport optical fiber with the transducer that converts the light signal from the PSD into an electric current. The removable cartridge connects to the acquisition unit, which has a circuit to integrate the electric current sent from the cartridge in a capacitor during an integration time. At the end of the integration time, the acquisition unit reads the charge accumulated in the capacitor, resets the capacitor, transforms that analog signal in a digital signal using an analog‐to‐digital converter (ADC) and sends the digital reading of the charge accumulated in the capacitor to the BlueSoft software, which plots the readings in real‐time.

**FIGURE 2:**
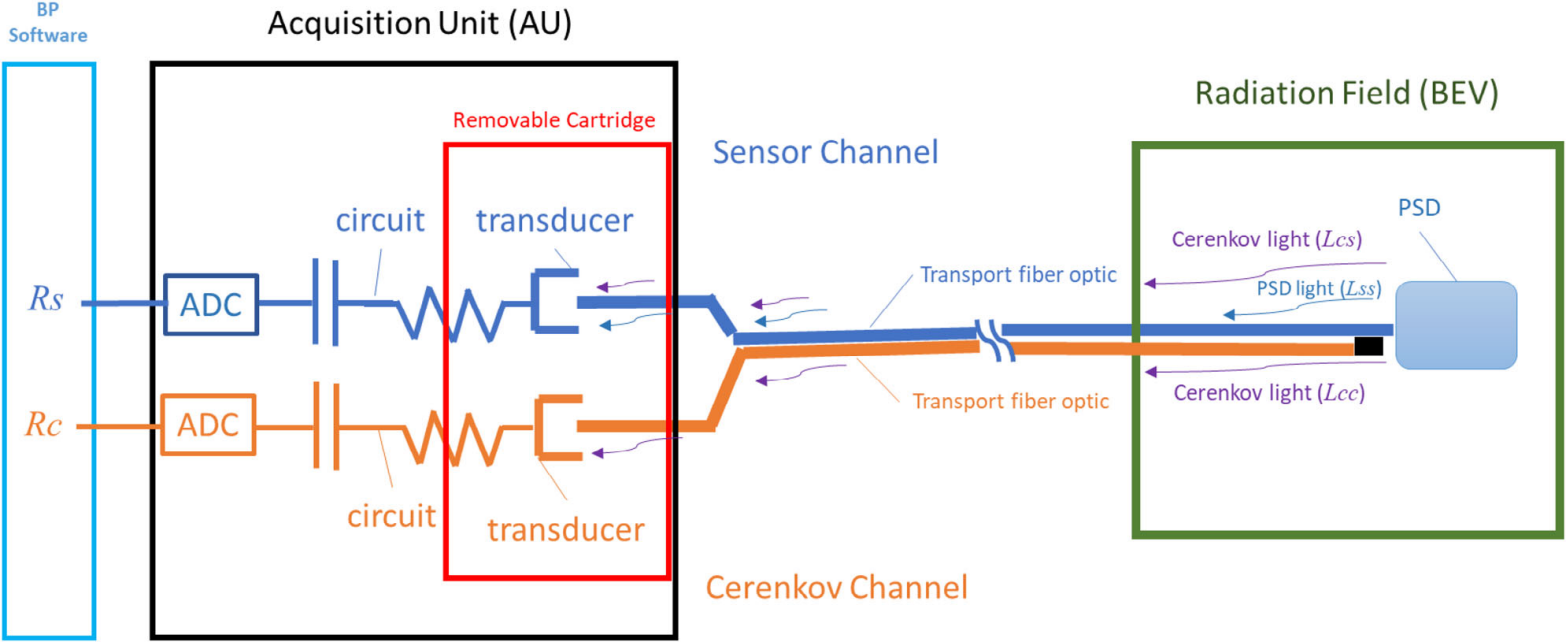
Schematic of Cerenkov subtraction process using dual channel optical fibers. Blue pathway is signal whereas orange pathway is Cerenkov signal. The signal is determined by *Rs‐Rc* and is electronically processed without user intervention.

The set composed by the PSD, transport optical fiber, transducer, capacitor and ADC is called the Sensor Channel. The readings for this channel, *Rs*, are expressed in nC. To subtract the Cerenkov radiation, BP‐PSD has a second channel called Cerenkov Channel which is identical to the sensor channel but it is not connected to the PSD. The transport optical fiber of the Cerenkov channel is set adjacent to the transport fiber of the sensor channel along its entire length. The readings from this channel, *Rc*, are also expressed in nC. This technique was previously described by Beddar et al.^17^ For PSD W2 detector, Jacqmin et al.^8^ provided a practical approach to subtracting Cerenkov radiation. A pictorial of Cerenkov subtraction for BP‐PSD is shown in Figure 2, indicating the position of sensor and Cerenkov Channel and the subtraction of the signal process. Most of these processes are performed via hardware with minimum intervention from the user. However, the Cerenkov subtraction process is elaborated below using large and small field method.

The Cerenkov radiation is a major problem in most PSD. Various techniques have been employed for correction which is needed for dosimetry. For example, Standard Imaging, W1 and W2 PSD use frequency discrimination (blue and green light) method as described by Guillot et al.^18^ and used in recent paper by Jacqmin et al.^8^ For BP‐PSD signal subtraction method as described by the manufacturer is used^19^ which is briefly described here. The amount of light produced by the PSD only is proportional to the dose deposited in the sensor. We can calculate the charge produced by this amount of light after subtracting the Cerenkov effect using this formula which is described elsewhere.^20^

(1) $$\mathrm{Signal}\;\left(\mathrm{proportional}\;\mathrm{to}\;\mathrm{Dose}\right)=\mathrm{Rs}-\mathrm{ACR}\times \mathrm{Rc}$$

(2) $$\mathrm{Dose}\;=\;\mathrm{CF}\times \left(\mathrm{Rs}-\mathrm{ACR}\times \mathrm{Rc}\right)$$

Where adjacent channel ratio (ACR) is the ratio between the two adjacent channels (sensor and Cerenkov), CF is calibration factor (cGy/nC) to convert the detector signal measured in nC to cGy. This is calculated after proper cross‐calibration under reference conditions using a calibrated ion chamber based on the code of practice.^21^, ^22^ ACR can be calculated as described by Underwood et al.^23^ Blue Physics describes this method, along with alternative methods, to calculate the ACR.^20^ There are various other methods to compute the ACR as described by Jacqmin et al.^8^ The method used in this study is the cross‐calibration method, which basically involves determining the ACR using the field output factor (FOF) of the 10×10 cm^2^ (*OF_10_*) and 3×3cm^2^ field (*OF_3_*).

(3) $$OF_{3}=\frac{\mathrm{Dose}\;\left(3x3\right)}{\mathrm{Dose}\;\left(10x10\right)}\;=\frac{Rs_{3}-\mathrm{ACR}\times Rc_{3}}{Rs_{10}-\mathrm{ACR}\times Rc_{10}}$$

(4) $$\mathrm{ACR}=\frac{OF_{3}\times Rs_{10}-Rs_{3}}{OF_{3}\;\times Rc_{10}-Rc_{3}}$$

Using Equation (4), ACR is calculated based on known FOF for the beam at the respective energy. All measurements were performed on a Varian TrueBeam unit that has been commissioned based on the TG‐106 guidelines.^24^ The scanning water tank was used for measurements, in conjunction with an image processing tool provided by BP‐PSD. Ion chamber measurements were also carried out to assess beam characteristics for comparison wherever needed. All measurement were performed for a field size of 10×10cm^2^ at 5 cm depth. Dose, dose rate, energy, angular dependence and small field dosimetry were carried out. The detector has 4 dots on detector at cardinal angles, thus for angular dependency test detector was rotated from one spot to the other. The BP‐PSD offers multiple data collection option, but one which is very attractive is fast mode that samples data based on pulses.

For small‐fields, various detectors but suitable for small‐field dosimetry such as the Standard Imaging PSD W2, and PTW microSilicon were also used to perform FOF at 95 cm SSD at 5 cm depth along with BP‐PSD. The characteristics of these detectors have been studied in small field.^3^, ^7^, ^8^, ^25^, ^26^, ^27^, ^28^, ^29^, ^30^, ^31^, ^32^ Data were collected with gantry angle of 0 degrees and 100 monitor unit (MU), with three readings per measurement taken for statistical analysis.

For temperature dependence, all detectors were connected to different fully calibrated electrometers for simultaneous measurements in a small water bucket (30×30×20 cm^3^) with variable temperature. The water tank was placed on 5 cm solid water phantom on treatment table. The gantry was rotated to 180 degrees to maintain the identical amount of attenuating materials in the beam, as the water quantity varied depending on addition and removal of water by adding hot water and later by ice. The temperature was varied from 5 to 45°C.

The depth dose and profiles of the photon beam were measured using a scanning water tank (IBA, Blue tank) at 100 cm source to surface distance (SSD). The field sizes were defined by the jaws and measurements were performed with ion chamber (0.125 cm^3^). The data were taken in both regular and fast mode of the BP‐PSD.

## 3 RESULTS

### 3.1 Dose linearity

**FIGURE 3:**
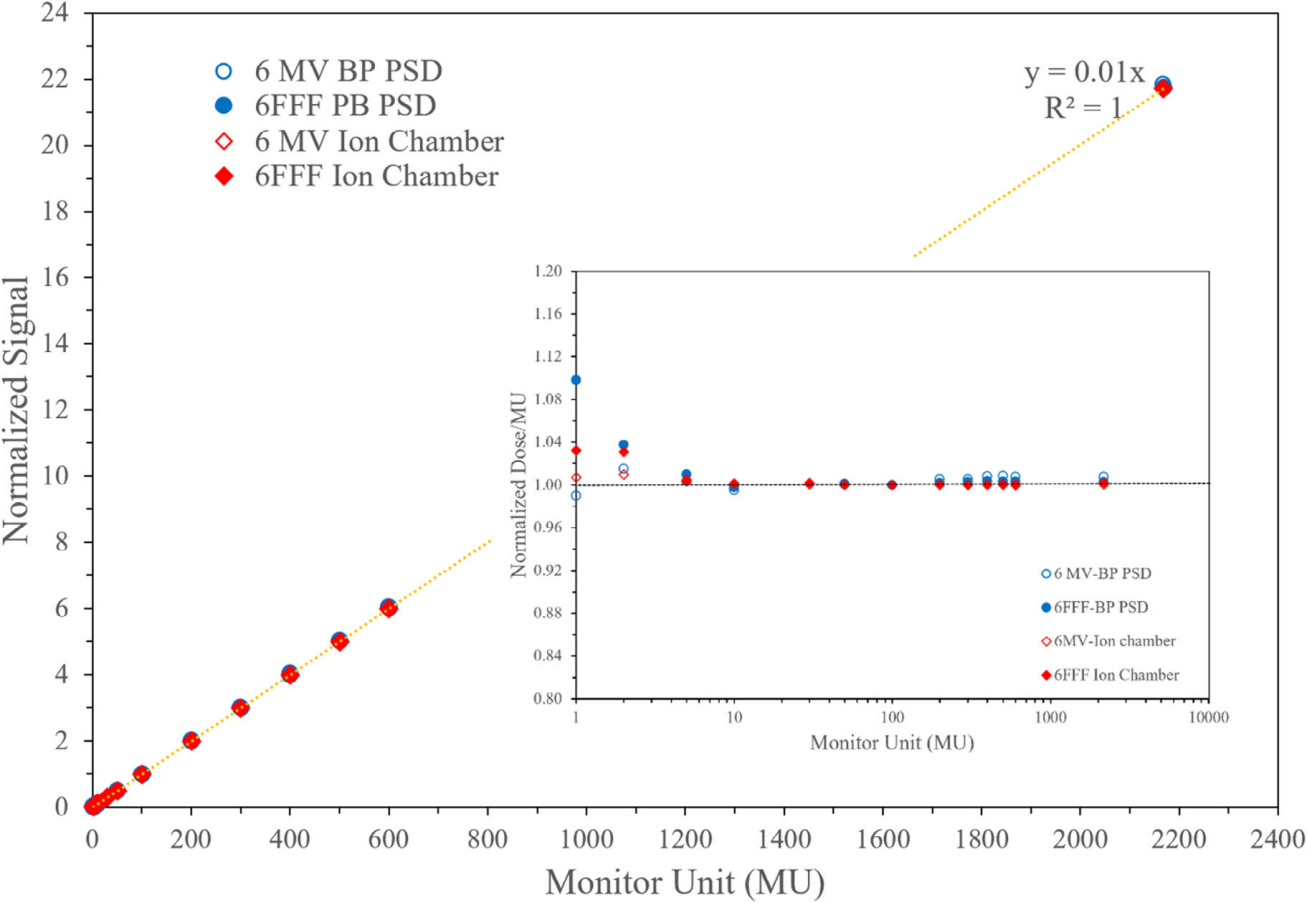
Dose linearity versus MU for ion chamber and BP‐PSD. Data for 6 MV and 6 FFF beams. Normalized dose/MU versus MU is plotted in the inset graph. Both ion chamber and BP‐PSD provided nearly identical data for MU > 2.

The dose linearity of the BP‐PSD was assessed over a wide range of MU for both 6 MV and 6 MV flattening filter free (FFF) beam. The data is plotted for the processed signal as well as the normalized signal/MU, as shown in Figure 3. The data demonstrates a linear relationship with R^2^ value of 1.0, indicating excellent linearity. However, when the data is plotted for Dose/MU, a slight nonlinearity is observed for low MU, although it still performs better than what has been shown in the literature for low MU.^33^, ^34^ The BP‐PSD data is compared with an ion chamber, and both show a similar trend for MU < 2 MU. Beyond 2 MU, the data becomes linear. This indicates that BP‐PSD can accurately provide dose linearity data for both low and high doses after proper Cerenkov radiation is subtracted, as shown in Equations (2)–(4).

**FIGURE 4:**
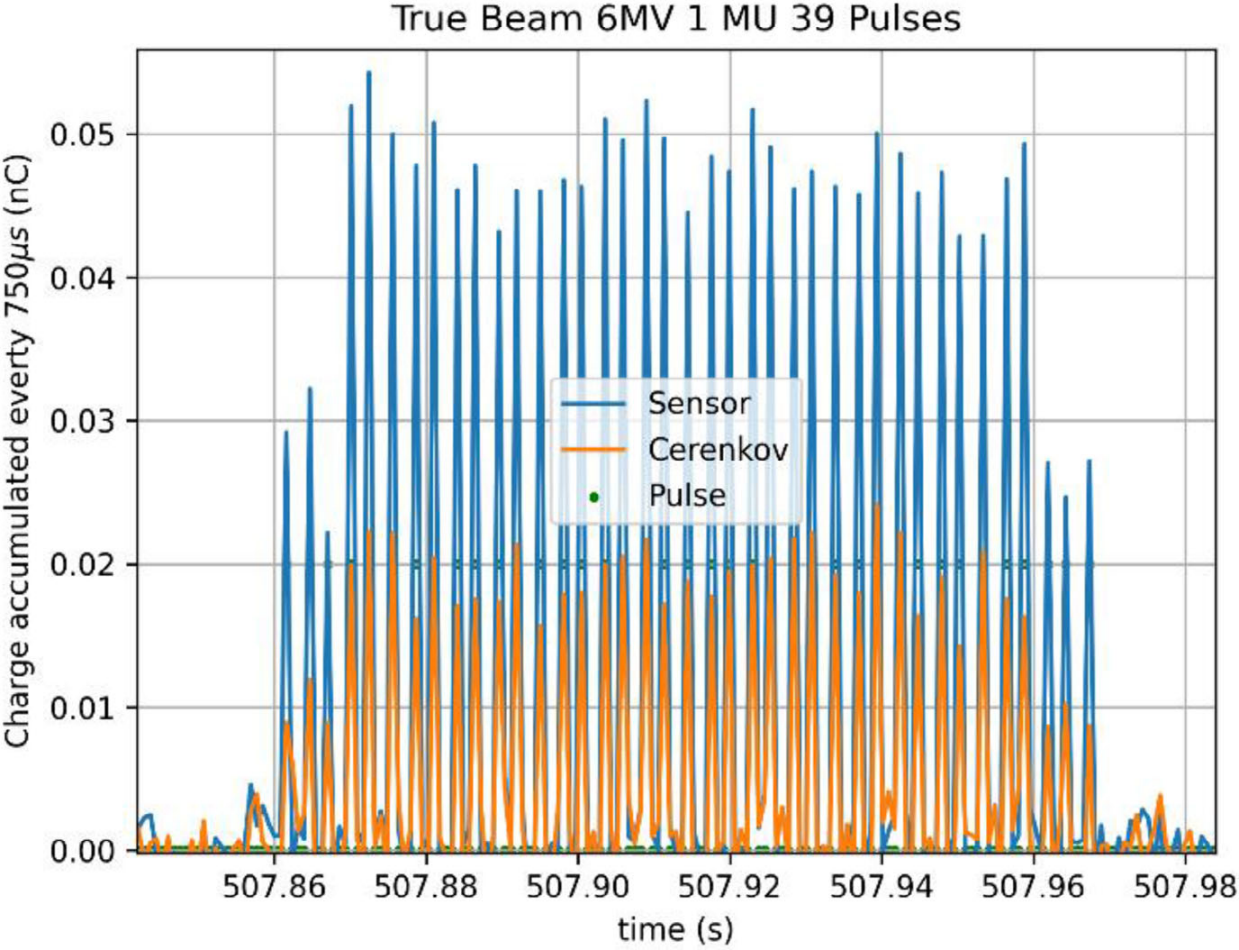
BP‐PSD data collected for 1 MU indicating sensor and Cerenkov readings. The system collects data in real‐time and provides the net results by subtracting the Cerenkov from the Sensor reading.

As mentioned earlier, the BP‐PSD utilizes pulse‐by‐pulse measurement or time‐dependent sampling at 700 μs, as shown for 1 MU in Figure 4. Small MU dosimetry has not been a concern in traditional dosimetry, as indicated in the references,^34^, ^35^ but it may be critical in adaptive therapy with multiple on/off beams where stability and ramping are critical. In such situation, pulse‐based dosimetry could provide meaningful dosimetry. The data in Figure 4 shows signals from the sensor and Cerenkov along with the beam pulse. The system can count the number of pulses in an MU if this is of importance to a clinical physicist.

### 3.2 Dose rate

**FIGURE 5:**
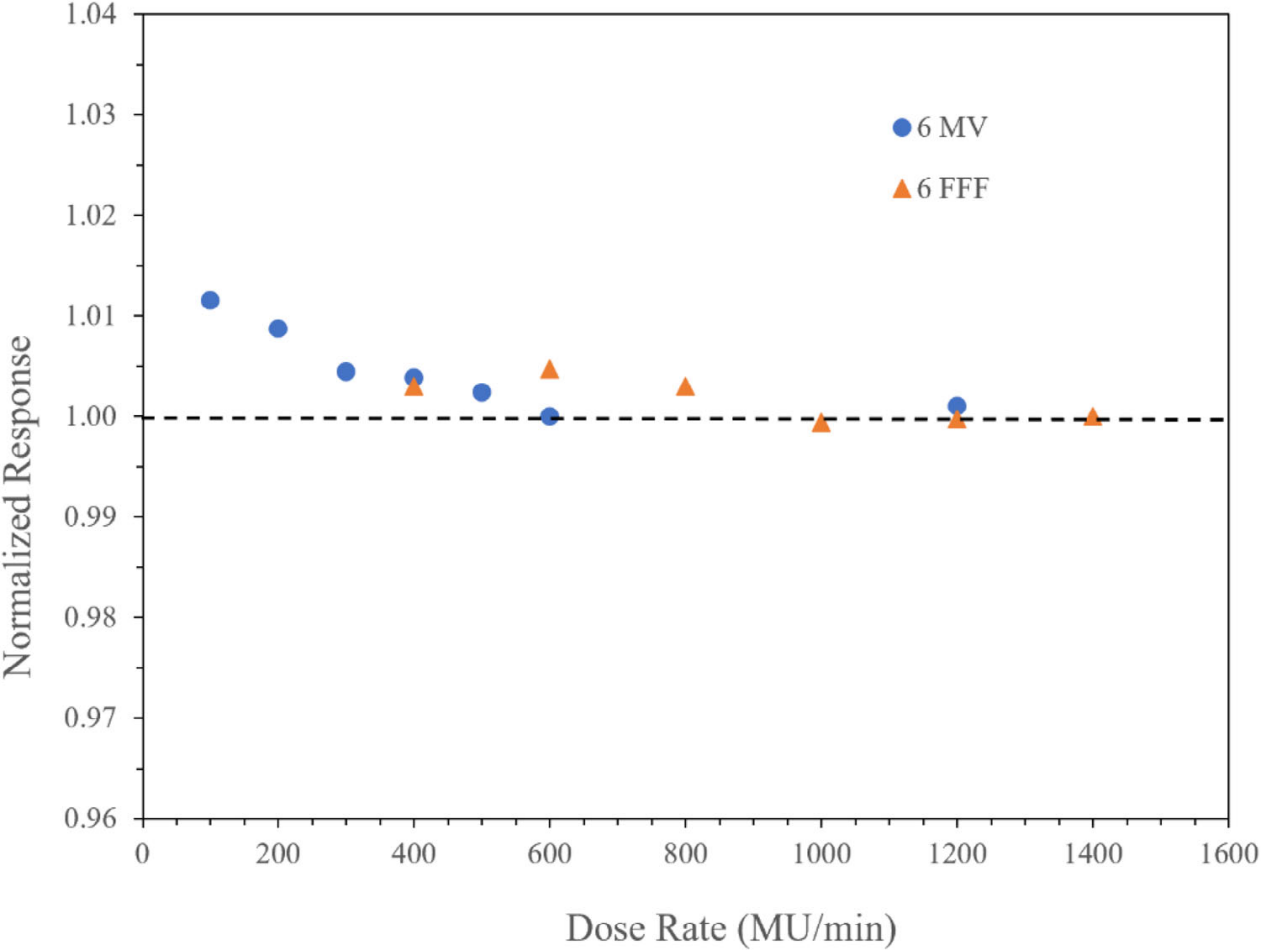
Dose rate response normalized to clinically used dose rate 600 MU/min and 1400 MU/min for 6 MV and 6FFF beam respectively. A line at 1.0 is drawn to show the difference in response.

Data was collected from 100 MU/min up to 1400 MU/min with BP‐PSD and ion chamber indicating linear response in 6 MV and 6 FFF beam. These data are normalized to respective clinically used dose rate; 600 MU/min and 1400 MU/Min for 6 MV and 6 FFF beams, respectively are shown in Figure 5. It shows that BP‐PSD can measure dose even in extreme ion recombination with high dose rate. The differences at low dose rate are <1%. The signal saturation in high dose rate is not visible in most clinical application. This aspect of the BP‐PSD could be tested in FLASH therapy where this system might provide advantageous.

### 3.3 Angular dependence

**FIGURE 6:**
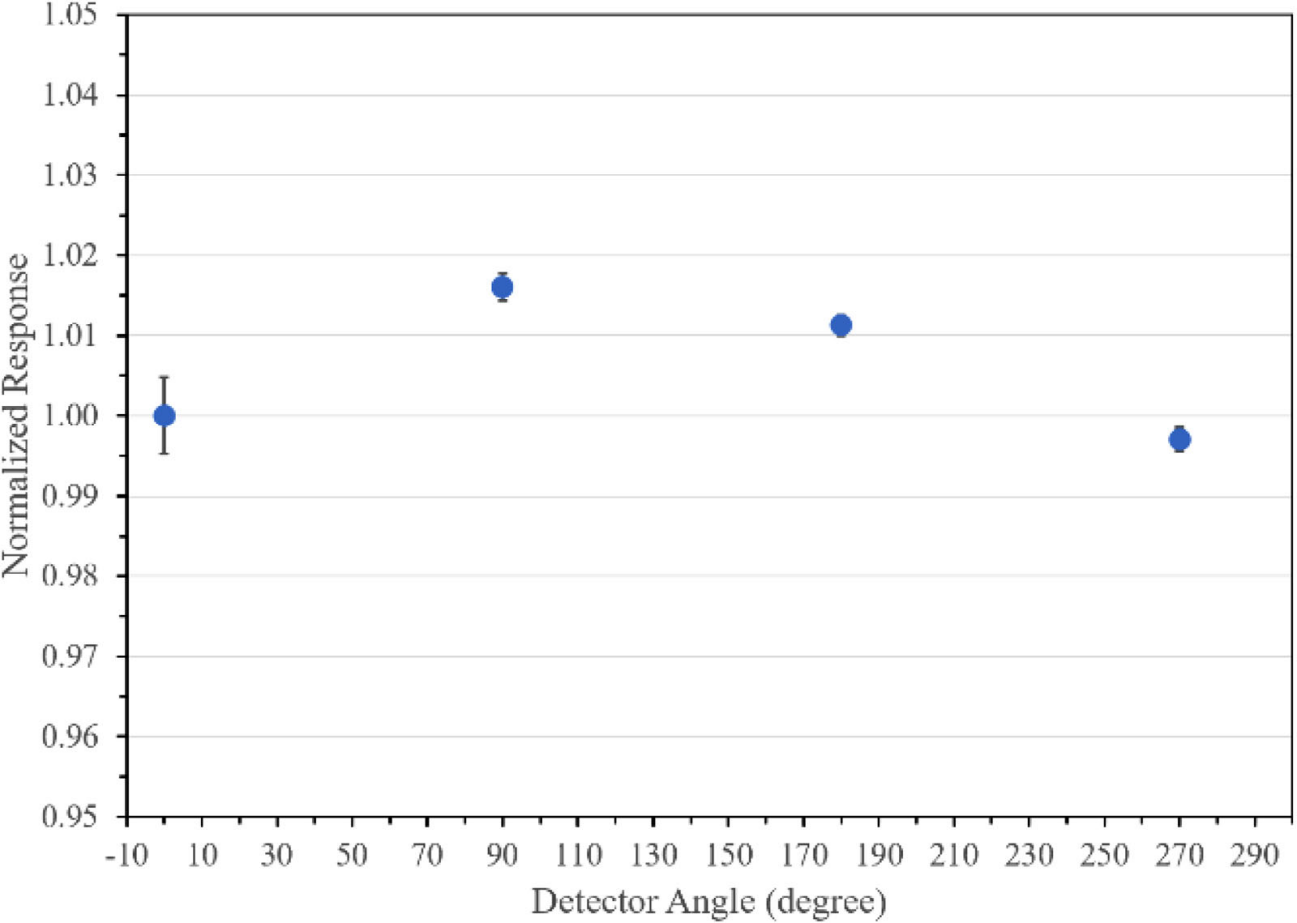
Angular dependence of the detector response.

The detector housing has four dots on cardinal angles. The data were collected by rotating the detector from one spot to the other in four cardinal angles (Figure 1a). The BP‐PSD response compared to zero degree is shown in Figure 6 indicating changes up to 1.5% difference at 90°. This may be due to housing materials that may not be symmetric and Cerenkov subtraction as shown in Figure 1 may not be same. Similar observation was also reported by Ferrer et al.^12^ and suggested that the PSD housing may not be symmetric. Additionally, a smaller detector size (under construction) might reduce this angular dependence but needs to be evaluated for other characteristics. However, it may be prudent to keep the detector in same orientation while collecting a set of data for reproducibility used in clinical settings.

### 3.4 Temperature dependence

**FIGURE 7:**
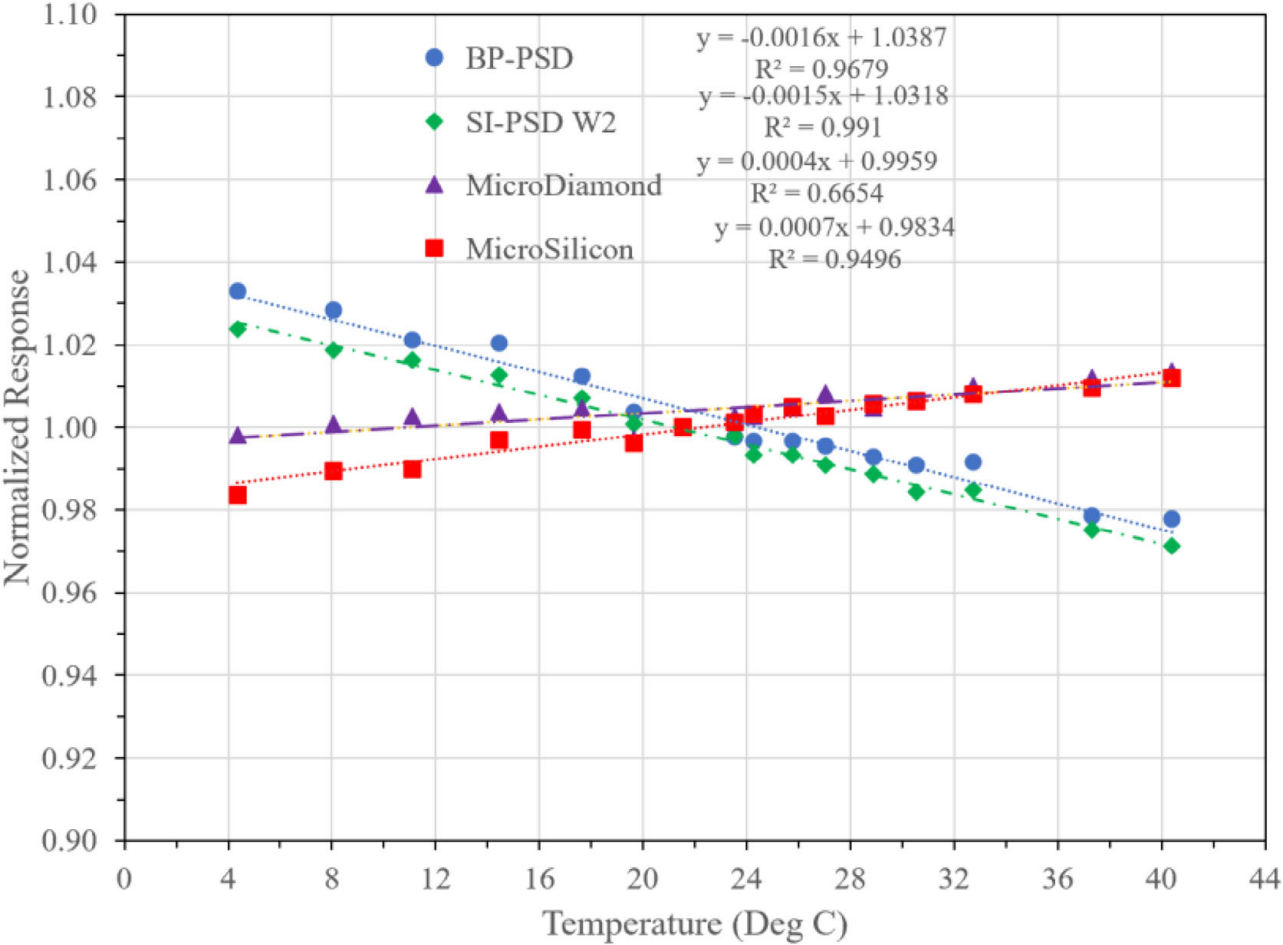
Temperature dependence of four detectors including BP‐PSD and Standard Imaging W2 normalized to 21.6°C. Regression lines are shown for each detector.

Temperature dependence is important factor which was evaluated as most detectors are used in phantom where temperature is variable. Unlike ion chambers where temperature and pressure correction can be applied based on ideal gas equation, solid state detectors need to be evaluated. Hence it was necessary to quantify the temperature dependence of BP‐PSD which was performed along similar microdetectors for comparisons. Figure 7 shows the data indicating subtle changes with temperature linearly. The PSD detector (W2 and BP‐PSD) response is downward trend with increasing temperature while other detectors show upward trend. It is important to emphasize that most clinical measurements are performed in a narrow temperature range. Most of these detectors are extremely stable and can be used. The variation in normalized response is less than ± 0.2% in the 18−24°C. This assures that BP‐PSD can be used in variable temperature with minimum degradation in data.

### 3.5 Depth dose and beam profiles

**FIGURE 8:**
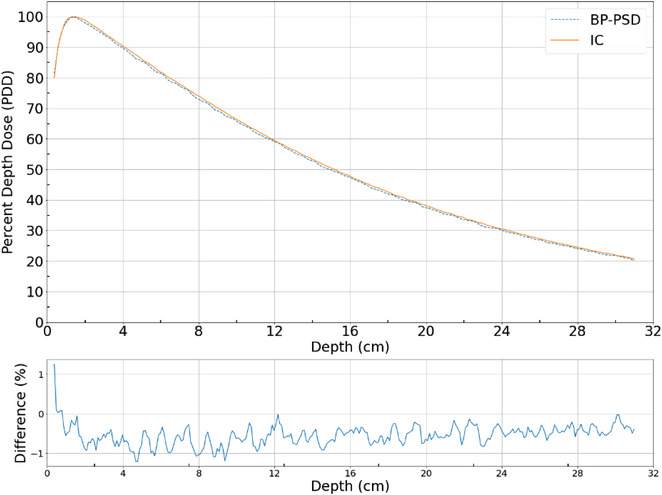
Depth dose of 6 MV beam taken with BP‐PSD and 0.125 cm^3^ scanning ion chamber. The difference plot is also shown between ion chamber and BP‐PSD.

**FIGURE 9:**
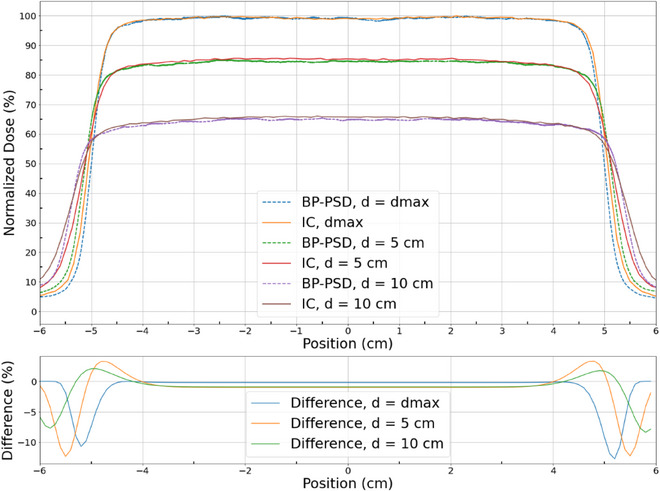
Profiles of 6 MV beam at dmax, 5 and 10 cm depths. The difference plot is also shown underneath indicating large variations.

Depth dose and profiles are highly sought raditation parameter of a machine that should be acquired accurately. This was measured in a water tank along with 0.125 cm^3^ scanning ion chamber as been described in TG‐106 with proper positioning.^24^ The data with BP‐PSD was acquired the same time after ion chamber measurements. The depth dose data from ion chamber and BP‐PSD are shown in Figure 8 and Profiles at d_max_, 5 and 10 cm depths are shown in Figure 9. The depth dose data for 6 MV are nearly identical <1% difference except in buidup region. The profiles are also very similar with difference plot which are <1% except in penumbra where these values differ up to 12%. Similar data were also collected for the FFF beam indicating similar patterns. The penumbral differences could be due to Cerenkov subtraction method which may not hold good and requires additional study.

### 3.6 Field output factor (FOF)

For small fields, ratio of readings cannot be equated to ratio of dose as shown by several publications^9^, ^10^ and require a correction factor as shown in Equation (5).

(5) $$FOF\;=\frac{D_{W,Q_{clin}}^{f_{clin}}}{D_{W,Q_{msr}}^{f_{msr}}}\;=\left[\frac{M_{Q_{clin}}^{f_{clin}}}{M_{Q_{msr}}^{f_{msr}}}\right]\;k_{Q_{clin,\;Q_{msr}}}^{f_{clin,}f_{msr}}$$

Where *D_w_* is the dose to water, *M* is detector reading and *k* is correction factor corresponding to a field size, *f*, for *clin* = clinical and *msr* = machine specific reference and *Q* is the beam quality. In small fields, the factor, $k_{Q_{clin,\;Q_{msr}}}^{f_{clin,}f_{msr}}$ has been a subject of great scientific interest with clinical implications for various microdetectors. A few of the detectors have *k* value ≈ 1.0 notably the plastic scintillator, microSilicon, and microDiamond with some exceptions.^3^, ^4^, ^7^, ^8^, ^23^, ^25^, ^28^, ^29^, ^31^, ^32^, ^36^ In this quest, BP‐PSD data compared to other detector is critical to verify that *k* ≈ 1.0 in that situation, this device can be used seamlessly in any radiation fields.

**FIGURE 10:**
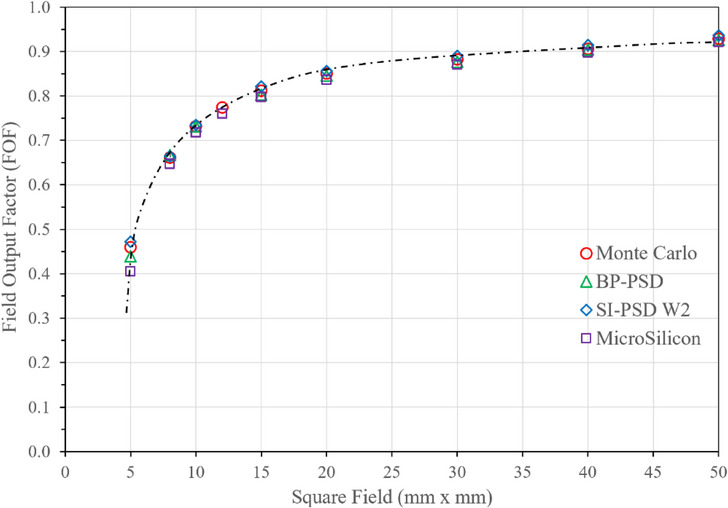
Field output factor (FOF) in small fields. Data are from various sources as shown in the refernces.

The PSD detector has shown to be a correction free detector in small field dosimetry.^9^, ^10^ Thus it was evaluated in small field in water phantom at 95 cm distance at 5 cm depth. Figure 10 shows the FOF data versus square fields. These data are not converted to *S_clin_* as has been shown to have minimal usage and geomtric field definition is more reproducible.^37^, ^38^ The data from detectors along with Monte Carlo (MC)^37^ are remarkable similar except for 5×5 mm^2^ field where the spread is relatively large (12%) which is due to setup and finite size of the detector. For BP‐PSD, FOF data was repeated 3 different days over 2 months. These data are reproduced very accurately with differnece ± 1.5% which cannot be plotted in error bars.

## 4 CONCLUSIONS

The PSDs are suitable detectors for radiation dosimetry especially in small field as they can be characterized as correction less detector (*k* ≈ 1.0). The difficulty with PSDs is the Cerenkov radiation that needs to be subtracted accurately. Future research in PSD could focus on developing improved Cerenkov radiation reduction, signal processing techniques and calibration methods to further enhance the accuracy and efficiency of PSD‐based dosimetry systems. Additionally, the BP‐PSD device gathers data on time scale, whereas most scanning systems move the device based on preset values. Currently, there is no direct connection between the BP‐PSD and any scanning water tank system, but this possibility is under consideration. The device mimics a thimble ion‐chamber, but smaller‐size PSD is available and can be used.

The BP‐PSD is a robust, reliable, and accurate detector that can be used in a scanning water phantom for radiation beam measurements in pulse‐by‐pulse mode. The data shown here are reproducible compared to ion chambers. Apart from extreme conditions, the detector can also be used for pulse‐by‐pulse dosimetry and, importantly, in small field dosiemtry, where the FOF data is in agreement with MC data, indicating that no correction is needed, which is typical of PSDs.

## AUTHOR CONTRIBUTIONS

All authors participated in data collection, analysis, writing and editing of the manuscript.

## CONFLICT OF INTEREST STATEMENT

None, except Marcos Feijoo is employee of the Blue Physics PSD system.
